# Effects of exogenous adiponectin supplementation in early pregnant PCOS mice on the metabolic syndrome of adult female offspring

**DOI:** 10.1186/s13048-020-00755-z

**Published:** 2021-01-18

**Authors:** Meng Zuo, Guotao Liao, Wenqian Zhang, Dan Xu, Juan Lu, Manhong Tang, Yue Yan, Chenghao Hong, Yuxia Wang

**Affiliations:** 1 Department of Reproductive Medicine, The First Affiliated Hospital, Jinan University, 601 West Huangpu Avenue, 510000 Guangzhou, People’s Republic of China; 2 The Second Hospital, University of South China, 421001 Hengyang, People’s Republic of China; 3 Department of Obstetrics and Gynecology, The Second People’s Hospital of Yueyang, 414000 Yueyang, People’s Republic of China

**Keywords:** Adiponectin, Polycystic ovary syndrome, Offspring, Metabolic syndrome

## Abstract

**Objective:**

PCOS is a heterogeneous endocrine disorder with both reproductive and metabolic abnormalities. At present, PCOS has been confirmed to have a certain genetic background. Compared with healthy women, the vast majority of PCOS patients have hyperandrogenemia, and this excessive androgen exposure during pregnancy may affect the development of female fetuses. The aim of the current study was to investigate the effect of adiponectin intervention during early pregnancy of obese mice with PCOS on the metabolic phenotype of adult female offspring.

**Methods:**

After the PCOS model was established, C57BL/6J mice were divided into maternal-control, maternal-PCOS, and maternal-PCOS + APN groups. DHEA-induced PCOS mice were supplemented with adiponectin (10 mg/kg/day) in the early pregnancy in order to eliminate adverse hormone exposure and then traced for endocrine indicators in their adult female offspring, which were observed for metabolism syndrome or endocrine disturbance and exhibited the main effects of APN. To further explore the underlying mechanism, the relative expressions of phosphorylated AMPK, PI3K, and Akt were detected in the ovaries of offspring mice.

**Results:**

The serum testosterone level of the maternal-PCOS + APN group in early pregnancy was significantly lower than that of the maternal-PCOS group (*p* < 0.01). The serum testosterone level in the offspring-PCOS + APN group was significantly lower than in the offspring-PCOS group (*p <*0.05), the diestrus time characterized by massive granulocyte aggregation in the estrus cycle was significantly shorter than in the offspring-PCOS group (*p*<0.05), and the phenotypes of PCOS-like reproductive disorders and metabolic disorders, such as obesity, insulin resistance, impaired glucose tolerance, and hyperlipidemia, were also significantly improved in the offspring-PCOS + APN group (*p *< 0.05). Compared with the control group, the expression levels of phosphorylated AMPK, PI3K, and Akt in the offspring-PCOS group were significantly decreased (*p *< 0.05), while those in the offspring-PCOS + APN group were significantly increased (*p *< 0.05).

**Conclusions:**

APN intervention in early pregnancy significantly reduced the adverse effects of maternal obesity and high androgen levels during pregnancy on female offspring and corrected the PCOS-like endocrine phenotype and metabolic disorders of adult female offspring. This effect may be caused by the activation of the AMPK/PI3K-Akt signaling pathway in PCOS offspring mice.

## Background

Polycystic ovary syndrome (PCOS) is one of the most common endocrine diseases in women of childbearing age with a prevalence rate of 10–18% [1, 2]. Clinically, it is characterized by hyperandrogenism, ovulatory dysfunction, and polycystic ovary morphology, often accompanied by insulin resistance and obesity [3–5]. Moreover, prevalence rates increase to over 25% in severely obese women with PCOS [6]. The etiology of PCOS has not been clarified yet, but, in recent years, more and more attention has been paid to the role of environmental and genetic factors in the occurrence and development of PCOS [7]. Family aggregation and twin studies have shown that PCOS has a strong heritable component [8]. However, the gene mutations identified so far cannot explain the high prevalence of PCOS in the population, which means that fetal exposure to environmental factors may also play an important role in the pathogenesis of this disease [9]. The interaction between heredity and sex hormones jointly affects the development of sexual dimorphism in mammalian offspring, while the poor nutritional status and inappropriate hormone levels of PCOS mothers during pregnancy may lead to metabolic disorders in offspring and permanently change the related endocrine phenotype of adult offspring [10–12]. Studies have pointed out that the exposure to adverse sex hormones during pregnancy and shortly after birth may affect the metabolism in adulthood. For such offspring of adult female rats, the testosterone marker showed obvious insulin resistance and metabolic dysfunction [13]. Eliminating the influence of inappropriate testosterone in the sensitive period is expected to be the key to normalize the endocrine phenotype of the offspring.

Adiponectin (APN) is a highly specific 30 kDa protein expressed by adipose tissue [14] and exists in human plasma at a high concentration (5–15 ug/mL, accounting for 0.01% of the total plasma protein) [15]. APN plays an important role in inhibiting the growth and metastasis of tumor cells because of its anti-proliferation effect and induction of apoptosis [16, 17]. In addition, APN improves the insulin sensitivity, is anti-inflammatory, and can participate in the prevention and treatment of diabetes and atherosclerosis [18, 19]. Insulin sensitivity is improved by APN through the cross-linking of multiple pathways. APN can induce extracellular calcium influx by activating AdipoR1 [20], which leads to the direct phosphorylation of AMP-activated protein kinase (AMPK) by calcium/calmodulin dependent protein kinase kinase2 (CAMKK2) in Thr172 [21, 22]. AMPK activated by APN can directly inhibit ribosomal protein S6 kinase 1 (S6K1); on the one hand, it reduces the risk of insulin resistance caused by increased phosphorylation of insulin receptor substrate-1 (IRS-1) in Ser636/639 [23], and, on the other hand, AMPK can also activate the phosphatidylinositol 3-kinase/protein kinase B (PI3K/Akt) signal transduction pathway for insulin signal transmission and regulation of the glucose metabolism [22, 24]. In addition, Akt can phosphorylate its own Ser485 or intermediate glycogen synthase kinase 3β (GSK3β) for negative feedback regulation of AMPK [25].

Some studies have shown that under the regulation of pregnancy-related molecules, the level of adiponectin in pregnancy is significantly higher than in non-pregnancy, while female patients with overweight or PCOS show generally a lower level of adiponectin than normal women [26], and they are more likely to suffer from metabolic diseases, such as gestational diabetes mellitus, during pregnancy [27, 28]. Based on the observation of the pregnant mouse model of APN deficiency, Qiao et al. found that pregnant mice with APN gene (Adipoq^−/−^) knockout showed abnormal glucose tolerance and hyperlipidemia in the third trimester of pregnancy, accompanied by fetal weight gain and abnormal offspring metabolism [29]. It is worth noting that these defects can be eliminated by adenovirus-mediated APN gene reconstruction during pregnancy to finally improve the quality of islet B cells.

Based on the important role of APN in regulating fatty acid oxidation and improving insulin resistance and the vertical effect of PCOS maternal endocrine environment on female offspring, this study focused on female offspring of obese mice with PCOS by observing the relevant metabolic phenotypes of each offspring after reaching adulthood to analyze the effect of APN supplementation during early embryo development on PCOS-related metabolic disorders in adult female offspring and further explore its mechanism.

## Methods

### Animal maintenance

All experimental procedures were approved by the Animal Ethics Committee of Jinan University. Immature female wild-type C57BL/6J mice (*n* = 60, four weeks old, 13 ± 1 g) were purchased from the Centre for Experimental Animals (Shandong, China). The animals were group-housed under standard laboratory conditions (room temperature: 22.5 ± 2.5 °C; relative humidity: 45.0 ± 2.0%; 12 h light/dark cycle with lights on at 8:00 am). Tap water and food pellets were provided ad libitum. The mice were kept for one week to acclimatize to the conditions and weighed daily during adaptive feeding and modeling.

Female mice were initially divided into (PCOS) model (*n* = 45) and control groups (*n* = 15). For modeling, the model group were given a high-fat diet (HFD: 60% fat content, China) [30] and subcutaneously injected with 0.6 mg/kg/day dehydroepiandrosterone (DHEA: Coolober Technology Co., Ltd., Beijing, China) [31] dissolved in 0.2 mL soy oil for 21 consecutive days. The control group were given a standard diet (SD:12 mm; Breeding Food, China) and subcutaneously injected with the same dose of 0.9% sodium chloride. After modeling, three mice in each group were randomly selected to verify successful modeling by the trends of body weight, serum testosterone level, and ovarian tissue section.

### Exogenous APN treatment in early pregnancy

After successful modeling, the model group (*n* = 39, three mice dead, three mice sacrificed) were randomly divided into two groups: maternal-PCOS group (*n* = 20; 0.9% sodium chloride, s.c.) and maternal-PCOS + APN group (*n* = 19; APN, 10 mg/kg/day, s.c.) [32]. The control group was recorded as maternal-control group (*n* = 10, two mice dead, three mice sacrificed; 0.9% sodium chloride, s.c.). Female mice received 10 IU pregnant mare serum gonadotropin (PMSG: ProSpec, Inc., Israel) and were injected with 10 IU human chorionic gonadotropin (HCG: LIVZON, Inc., China) 48 h later. Females of the different groups were randomly mated with untreated C57BL/6J wild-type males (8 weeks old, 25 ± 2 g). Mice with vaginal plug were recorded at day 0 of pregnancy.

Adiponectin (APN: gAcrp30, murine recombinant, BioVision, USA) was dissolved in Tris solution (1 mL, 5 mM, pH 7.6), and this stock solution was used to prepare an APN solution with a concentration of 0.1 mg/mL. APN injection with this solution began on day three after pregnancy, and treatment of the maternal-PCOS + APN group was continued for 11 days. After completion of the injection, three female mice in each group were randomly selected to obtain blood samples for the detection of the levels of serum androgen and adiponectin.

**Fig. 1:**
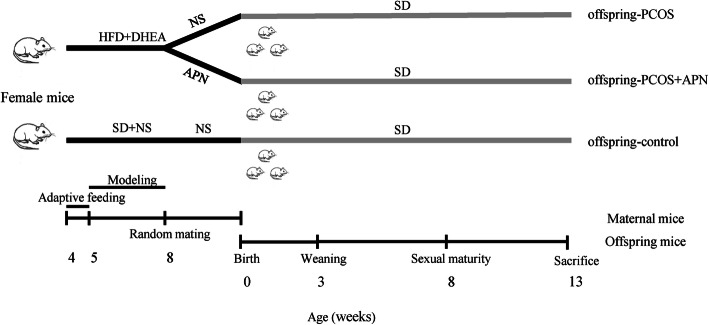
Schematic representation of the different experimental groups: offspring-control group (*n* = 6; maternal-control group offspring, SD); offspring-PCOS group (*n* = 6; maternal-PCOS group offspring, SD); offspring-PCOS + APN group (*n* = 6; maternal-PCOS + APN group offspring, SD)

The pregnancy rate was quantified per treatment and pairing throughout the observation process. Pregnant mice were assigned to the corresponding groups at the time of weaning (Fig. 1). The standard diet was given to all mice during breeding, lactation, and growth of the young stock. The nutritional profile of the standard diet was the following: protein 20%, fat 4%, fiber 5%, ash 8%, moisture 10%, nitrogen-free extract 50%; calories: 3.78 kcal/g.

### Phenotypic indices and vaginal cytology

Bodyweight (BW) and food intake of offspring mice were monitored daily from weaning (3 weeks old) to slaughter (13 weeks old). In adulthood (8 weeks old), vaginal smears were performed daily for 20 consecutive days (4–5 cycles) [33]. The estrous cycle of offspring mice was monitored by vaginal smears and assessed by optical microscopy.

### I.p. Glucose Tolerance Test (IPGTT) and I.p. Insulin Tolerance Test (IPITT)

IPGTTs were performed of non-fasted offspring mice (8 weeks old), and their food was removed 16 h before the experiment. One week after IPGTT, IPITTs were performed of mice that were fasted for 6 h. Tests were performed by i.p. injecting mice with either glucose or insulin, as previously described (IPGTT: 10% glucose, 0.5 g/kg BW; IPITT: insulin 2 U/kg BW). The blood glucose concentration of tail vein blood was measured with a glucose monitoring kit (Johnson, USA) at 0, 15, 30, 60, 90, and 120 min after glucose or insulin injection.

### Tissue and blood sample collection

At the end of the experiment, all offspring mice (13 weeks old) were anesthetized, blood samples were obtained by eyeball enucleation, and food was removed overnight for a maximum of 8 h (water was unlimited) before slaughter. The serum was immediately separated and stored at − 20 °C for subsequent analyses. After collection of blood samples, the bilateral ovaries of the mice were rapidly removed, and some of the fresh ovarian tissue samples were rapidly frozen at − 80 ℃ for biochemical analysis. The other part of the ovaries were fixed in 4% paraformaldehyde solution. After embedding in paraffin, the ovaries were cut into 5-µm thick sections and stained with hematoxylin-eosin (H&E), and the tissue structure was observed by electron microscopy.

### Biochemical analyses and serum hormone measurements

Fasting blood glucose (FBG), total cholesterol (TC), triglycerides (TG), HDL cholesterol (HDL-C), and LDL cholesterol (LDL-C) levels in the serum were measured using an autoanalyzer (BS-5800M, Mindray, China). The homeostasis model assessment-insulin resistance (HOMA-IR) index was calculated as previously described [34]. Serum insulin (INS), adiponectin (APN), and testosterone (T) concentrations were measured using an ELISA kit (CUSABIO, Wuhan, China) according to the manufacturer’s instructions.

### RNA extraction and real-time PCR

Total RNA was extracted from the ovarian tissue of offspring mice with the TRIzol reagent (Invitrogen) to synthesize first-strand cDNAs by using the EasyScript TM First-strand cDNA Synthesis SuperMix (TransGen Biotech, China). Quantitative real-time PCR (qRT‐PCR) was performed to determine the total cDNA with a 7500 real‐time PCR system (Applied Biosystems, USA). The qRT‐PCR was performed using a ImProm-II™ Reverse Transcription System (Promega, USA). Specific primers for amplification of specific genes were purchased from Sangon Biotech (Shanghai, China), as listed in Table 1. Expression levels were determined by the 2^-ΔΔCt^ method, Cicated as the ratio with respect to the control.

**RT‐ PCR primer sequences**

**Table:** 

Primer name	Nucleotide sequence (5′‐3′)
AKT- forward	CAAGGCCCAACACCTTTATC
AKT- reverse	ACGATGACCTCCTTCTTGAG
AMPK- forward	TGTAGAGCAATCAAGCAGTT
AMPK- reverse	TCCTTTGGCAAGATCGATAG
PI3K- forward	GGGCAGTTAAGAAGCACAATG
PI3K- reverse	GCAGGAGAGTCTTTCCAATG
GAPDH- forward	GGCCTCCAAGGAGTAAGAAA
GAPDH- reverse	GCCCCTCCTGTTATTATGG

### Western blotting

Protein was extracted from ovarian tissue of offspring mice. Total protein concentrations were measured using a BCA protein assay kit (Solarbio, China). Equal amounts of proteins (15 µg) were resolved by SDS-PAGE and then electrophoretically transferred to PVDF membranes. Subsequently, the membrane was blocked with 5% BSA (Solarbio, China) for 2 h and probed with different primary antibodies at 4 °C overnight. The main primary antibodies were phospho-AMPKα (Thr172; CST, USA), phospho-PI3 kinase p85(Tyr458)/p55(Tyr199) antibody (CST, USA), phospho-Akt (Ser473) antibody (CST, USA), phospho-Fox03a (Ser253) antibody (CST, USA), caspase-3 antibody (CST, USA), and Fox03a antibody (Abcam, UK). After thoroughly washing three times with TBST for 5 min each time, HRP‐linked secondary antibody (CST, USA) was used to detect the primary antibodies, followed by an additional 1-h incubation at room temperature. At last, bands were visualized using an Amersham Imager 600 (GE, USA), and band intensities were analyzed using the computer Image J 6.0 software (National Institutes of Health, USA).

### Statistical analyses

All data were expressed as the mean ± SD and analyzed using the GraphPad Prism 8.0 (GraphPad Software, Inc., USA) and SPSS 25.0 (SPSS, Inc., Chicago, IL, USA). The Wilcoxon rank-sum test was used to compare two independent samples of the original data, and the Kruskal-Wallis test was used for non-normally distributed data. Multiple-group comparisons were evaluated by one-way ANOVA with post-hoc testing. Tests were performed within the 95% confidence interval, and the significance level was set at *p* < 0.05.

## Results

### Successful establishment of PCOS model and offspring breeding

**Fig. 2:**
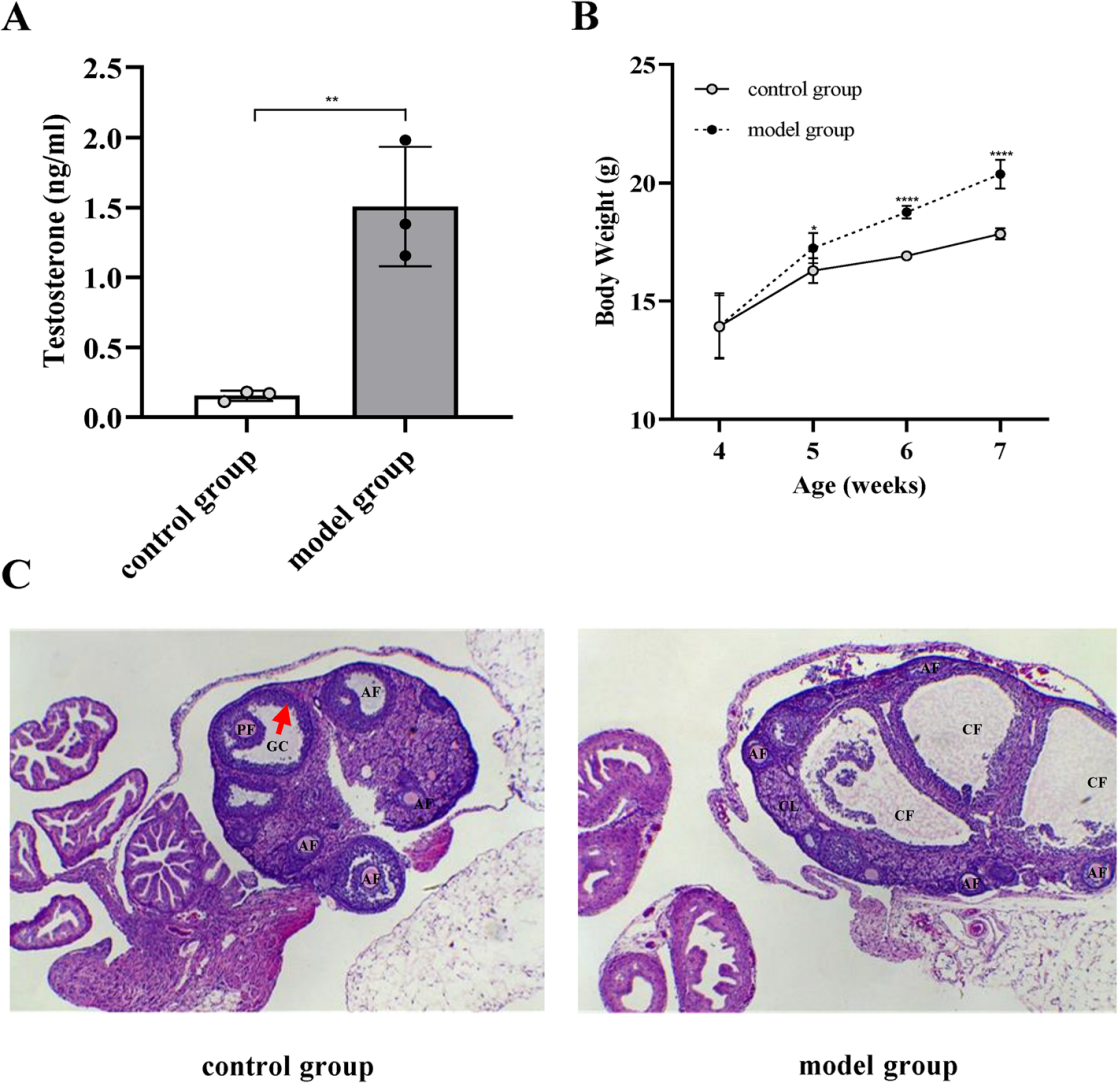
Establishment of the PCOS model. **a** Changes in the active testosterone expression in mice after an effective dose of DHEA stimulation; **b** significant differences in the body weight between the control and model groups; **c** ovarian tissue sections were prepared from control or model mice for H&E staining. Antral follicle (AF), preovulatory follicle (PF), cystic follicle (CF), granulosa cell (GC), and corpora lutea (CL) were annotated. ^*^p < 0.05, ^**^p < 0.01, ^***^p < 0.001, ^****^p < 0.0001 versus untreated control mice. Data are indicated as mean ± SD (*n* = 3)

The difference between the weight gain of mice in the model and control groups was statistically significant (*p *< 0.05; Fig. 2b). The serum testosterone level in the PCOS model group was significantly higher than in the control group (*p *< 0.01), which is consistent with the symptoms of hyperandrogenemia in PCOS (Fig. 2a). Atresia and cystic dilated follicles were significantly increased in the model group compared with the control group. Moreover, the radiating crown disappeared, the granulosa cell layers became loose, and their number decreased. However, multiple follicles at different developmental stages were detected in the ovaries of mice in the normal control group (Fig. 2c). This indicates that the mouse PCOS model was successfully established. In addition, PCOS adult female mice also showed impaired fertility, as indicated by significantly fewer litters, showing significant differences in the pregnancy rate (χ2 = 11.57, *p < *0.01) and average litter size (F = 8.85, *p < *0.01) among different groups of maternal mice (Table 1).

**Table 1:** Fertility indexes of mice of each group (mean ± SD)

Groups	Total (pcs)	Pregnancy (pcs)	Pregnancy rate (%)	Average litter size (pcs)
maternal-control	7	6	85.71	7.00 ± 2.28
maternal-PCOS	17	5	29.41^*, ++^	1.80 ± 0.30 ^**, +^
maternal-APN + PCOS	16	13	81.25	4.08 ± 2.18^*^

^*^, ^**^ indicate significance of the maternal-control group at *p* < 0.05 and *p* < 0.01 probability level, respectively; ^+^, ^++^ indicate significance of the maternal-PCOS + APN group at *p* < 0.05 and *p* < 0.01 probability level, respectively

### Prenatal hyperandrogenic exposure triggers endocrine disorders similar to PCOS in adult female offspring

To test whether exposure to a high androgen environment during pregnancy leads to a PCOS phenotype in adult offspring, we simulated hyperandrogenic exposure in female mice during pregnancy after establishing the PCOS model and studied endocrine and reproductive features of female offspring in adulthood.

The serum androgen level of the maternal-PCOS group was higher than that of the maternal-control group (*p *< 0.01), and the maternal-PCOS group exhibited more obvious hypoadiponectinemia than the maternal-control group (*p < *0.05). After APN intervention in early pregnancy, hyperandrogenemia and hypoadiponectinemia of maternal-PCOS mice were significantly improved.

**Fig. 3:**
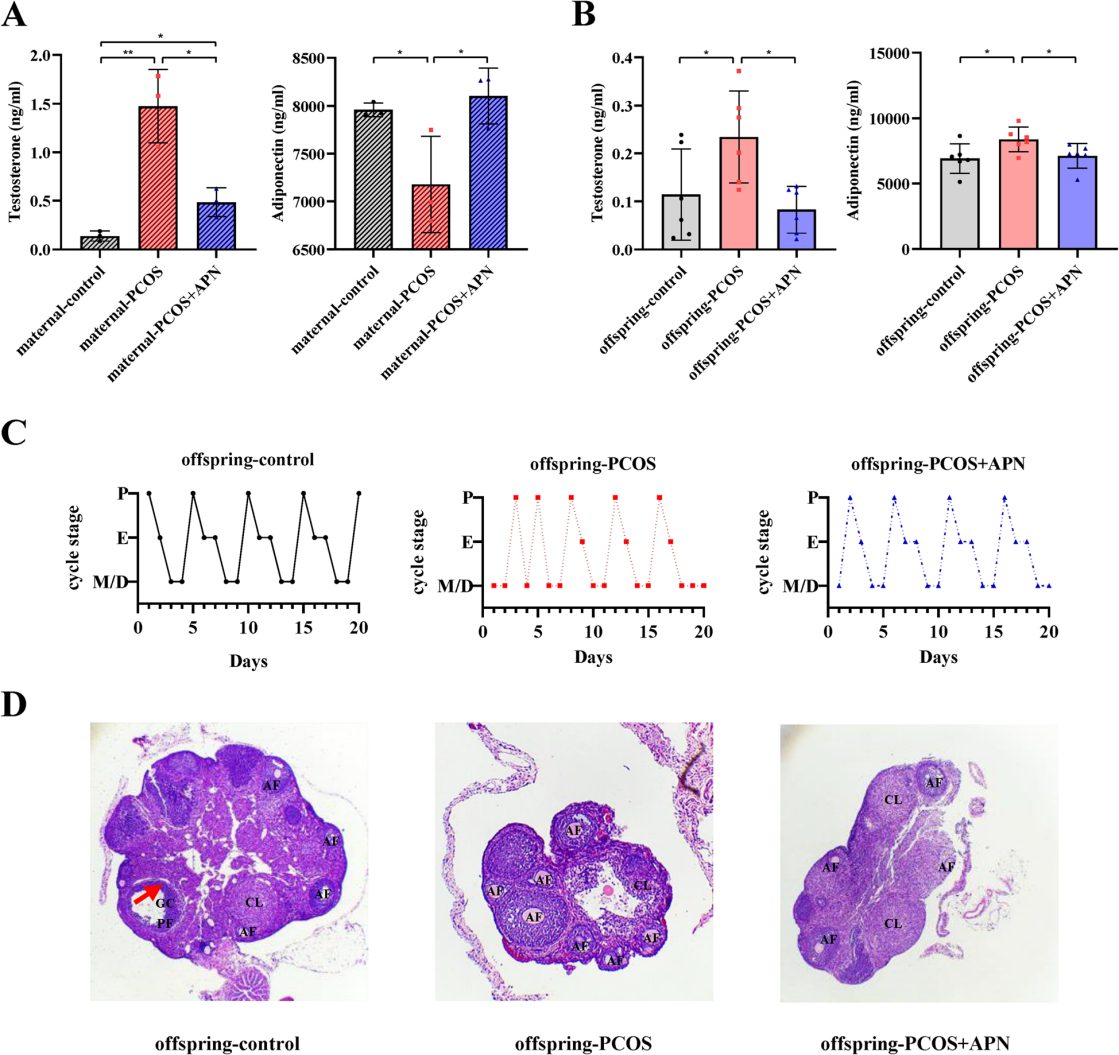
Hormone levels in trunk blood measured by ELISA for each group. Maternal (**a**) and offspring (**b**) serum adiponectin and testosterone levels were determined. (**c**) Representative estrous cycles of six mice per group during 20 consecutive days. (**d**) Representative photomicrographs of ovaries stained with hematoxylin-eosin of control, PCOS, and PCOS + APN mice. Antral follicle (AF), preovulatory follicle (PF), cystic follicle (CF), granulosa cell (GC), and corpora lutea (CL) were annotated. ^*^p < 0.05, ^**^p < 0.01

**Fig. 4:**
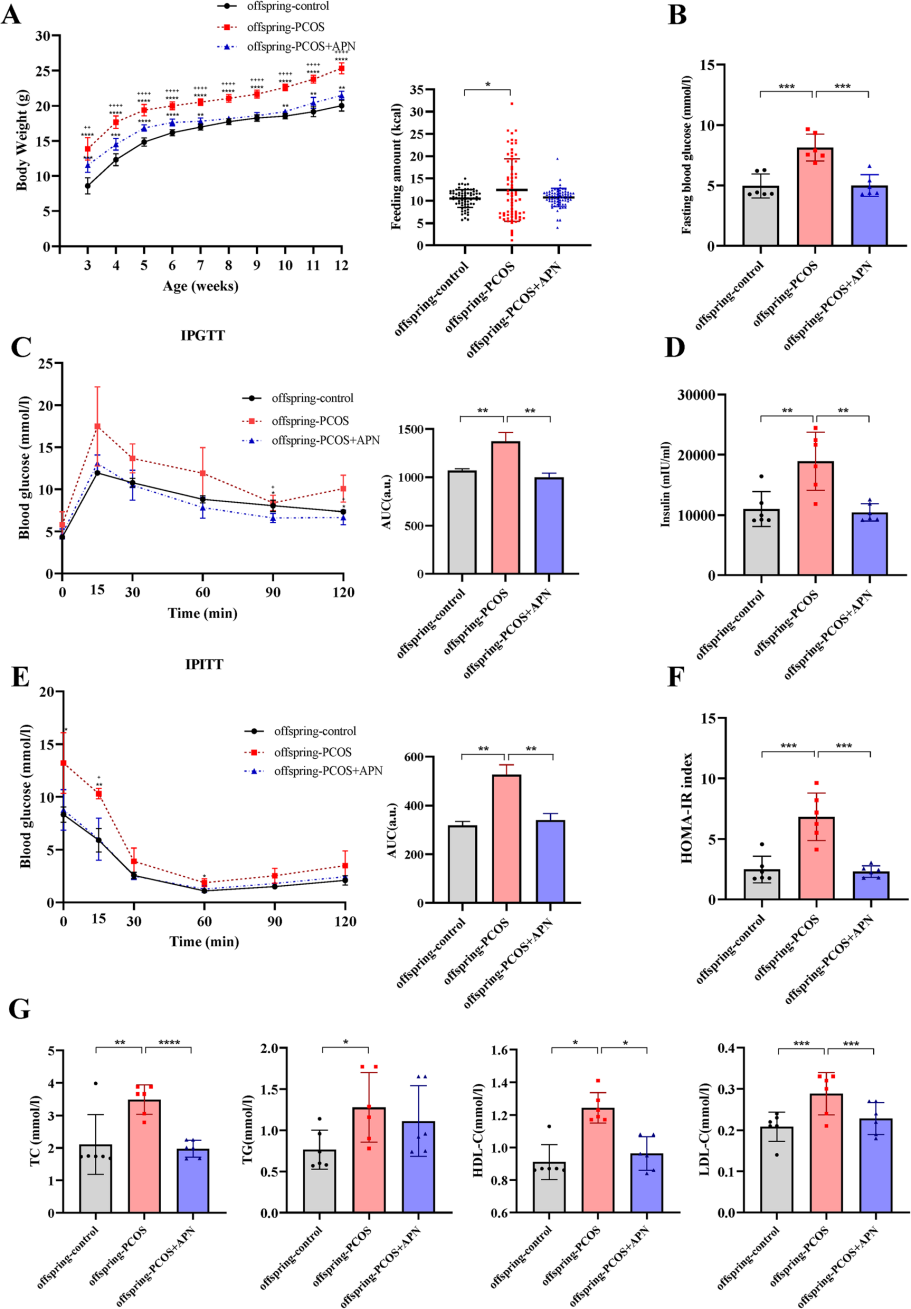
Adiponectin improves serum lipid profiles and the glucose metabolism of PCOS offspring mice. Antidiabetic effect of adiponectin on PCOS offspring mice: BW (**a**), basal blood glucose (**b**), IPGTT and AUC levels (**c**), IPITT and AUC levels (**e**), serum insulin (**d**), and HOMA-IR index (**f**). **g** TC, TG, HDL-C, and LDL-C levels of serum that was obtained from 13-week-old mice of each group

Moreover, PCOS offspring showed weight gain, eating disorders, hyperandrogenemia (*p* < 0.05; Figs. 3 and 4c and a), and changes in the estrous cycle. Compared with the offspring-control group, offspring-PCOS mice rarely entered the preovulatory stage of the estrous cycle and displayed prolonged time in metestrus/diestrus (Fig. 3d). Ovarian histology of PCOS progeny showed consistent abnormalities. Compared with the offspring-control group, the number of corpus luteum was significantly reduced after ovulation, and no dominant follicles appeared (Fig. 3e). Phenotypic characterization of the offspring (Fig. 4b) showed that prenatal hyperandrogenic exposure triggers endocrine disorders similarly to PCOS in offspring mice, which seems to be effectively improved by maternal APN intervention in early pregnancy.

### Adiponectin therapy in early pregnancy of PCOS mice normalizes the endocrine phenotype of adult female offspring

After successful modeling, pregnant mice of offspring-control and offspring-PCOS groups were subcutaneously injected with 0.9% saline, and the offspring-PCOS + APN group was treated with APN (10 mg/kg/day) during early pregnancy (P3–P13, 11 days) within the same temporal window.

Observation of the metabolisms of offspring-PCOS and offspring-PCOS + APN groups showed that the PCOS-like neuroendocrine phenotype and metabolic disorders of female offspring treated with APN during early pregnancy tend to normalize. Compared with the offspring-PCOS group, the fasting levels of blood glucose and insulin were decreased (Fig. 4b, d), impaired glucose and insulin tolerance were alleviated, and insulin sensitivity was improved in the offspring-PCOS + APN group (Fig. 4c, e). In addition, compared with the offspring-PCOS group, total cholesterol and low-density lipoprotein levels in the serum of the offspring-PCOS + APN group were significantly decreased (Fig. 4f) and approached the levels of offspring-control mice. Surprisingly, the offspring showed completely opposite results at the adiponectin level compared with mother mice. Compared with APN-treated offspring, the adiponectin level of PCOS offspring was significantly higher (Fig. 3a, b).

### Effects of adiponectin on the activities of phosphorylated AMPK, PI3K, and Akt in the ovarian tissue of offspring mice

Protein kinase activities in the ovaries were assessed by detecting the phosphorylated forms of AMPK, PI3K, and Akt and further determined based on changes in the mRNA of the cells to investigate whether AMPK, PI3K, and Akt might be responsible for the protective effect of adiponectin.

**Fig. 5:**
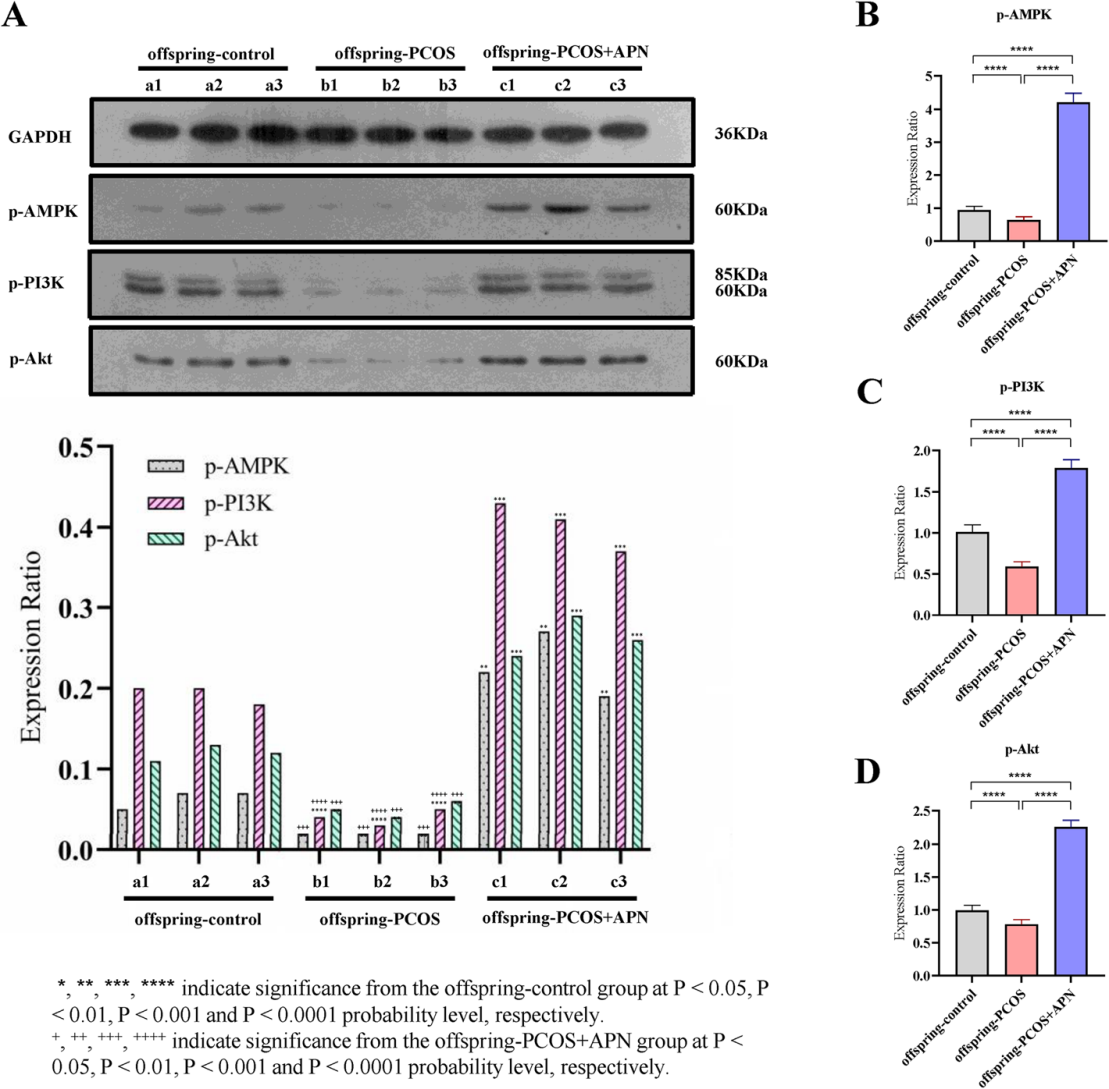
Effects of adiponectin on AMPK, PI3K, and Akt phosphorylation expression in PCOS offspring mice. **a** Immunoblotting of protein extracts from ovarian tissues of PCOS offspring mice treated with adiponectin during the embryonic phase in comparison with control and untreated PCOS offspring mice. **b-d** Content of mRNA of phosphorylated AMPK, PI3K, and Akt of mice of each group

As shown in Fig. 5a, compared with the offspring-control group, subcutaneous injection of adiponectin during early pregnancy significantly increased the levels of AMPK, PI3K, and Akt phosphorylation in ovarian tissue, while the phosphorylation levels of the offspring-PCOS group were significantly decreased. In addition, the mRNA content of phosphorylated AMPK, PI3K, and Akt was measured in the cells, revealing a consistent trend with changes in the protein phosphorylation levels (Fig. 5b–d). APN treatment in early pregnancy had a significant impact on the production of phosphorylated AMPK, PI3K, and Akt in the offspring’s ovaries.

## Discussion

Inappropriate hormone exposure during the early pregnancy, which is a sensitive period for different organizations or systems, including the central nervous system, heart, liver, and reproductive organs, can induce large metabolic alterations in adult life, and most women with PCOS have unexplained hyperandrogenic levels during pregnancy [35, 36]. Cimino I et al. confirmed that the increased binding of GABA and GnRH neurons in the offspring caused by hyperandrogenemia during pregnancy. Persistent overactivity of GnRH neurons in the offspring after adulthood leads to great changes in HPO axis reactivity and sex hormone levels [33]. These changes activate the immune system of the offspring, which in turn leads to a chronic inflammatory response characterized by reproductive endocrine disorders [33, 37]. Meanwhile, Epidemiological analysis has demonstrated links between maternal obesity and offspring disease. The risk of organ deficits and related diseases caused by obesity has circular programming in subsequent generations [38]. In the current study, we have demonstrated that exposure to obesity and high-dose testosterone during pregnancy can cause severe metabolic disorders in adult female mice of PCOS offspring, mainly manifested by hyperandrogenemia and metabolic disorders, which centered on insulin resistance. In addition, APN intervention in early pregnancy could significantly improve the hyperandrogenemia and hypoadiponectinemia of obese maternal-PCOS mice, thus significantly improving the reproductive metabolic phenotype of offspring.

Antenatal adverse events alone may not be sufficient to determine the occurrence of PCOS in the offspring, and adverse stimuli (endocrine imbalance and/or environmental exposure) during subsequent life after birth may reveal or amplify potential defects associated with PCOS [39]. Anderson AD et al. reported that obesity can increase the androgen production in the ovaries/adrenal glands through insulin resistance and compensatory hyperinsulinemia and inhibit sex hormone-binding globulin (SHBG) to increase the bioavailability of androgen. Both effects reinforce each other, aggravating the development of PCOS [40, 41]. Our data indicate that androgen exposure during pregnancy and severe eating habit disorders are significant factors leading to obesity in PCOS offspring. Under the premise that the average daily food intake is similar, the weight of PCOS offspring is significantly higher than that of normal offspring, and the persistent obesity during the growth of the offspring further increases the risk of hyperandrogenism in adulthood. Based on clinical observations, Sir-Petermann et al. found that part of the metabolic characteristics of PCOS existed already in the early childhood of the daughters of PCOS patients. In contrast, the levels of serum insulin and triglyceride further increased and the level of serum SHBG decreased in the puberty of PCOS offspring, and hypoadiponectinemia occurred much earlier than hyperandrogenemia [42]. It has been confirmed that APN has a considerable degree of sensitivity and is an early sign of metabolic disorder in PCOS offspring. We found that maternal PCOS pregnant mice also showed a considerable degree of hypoadiponectinemia compared with normal pregnant mice, and the serum APN concentration of PCOS pregnant mice reached the normal level after APN supplementary intervention, which reduced the hypoadiponectinemia environmental exposure caused by hyperandrogenic and obesity of PCOS female offspring in the embryonic stage to a certain extent.

On the contrary, based on the above conclusions, we found that after adiponectin treatment, a series of metabolic disorders, such as insulin resistance, impaired glucose tolerance, and elevated blood lipids, were improved in PCOS offspring. In addition, the serum testosterone level of the offspring-PCOS + APN group was significantly lower than that of the offspring-PCOS group after reaching adulthood, which confirmed that the embryonic APN intervention treatment corrected the high androgen level of PCOS female offspring, reduced inflammatory reactions in the reproductive tract, and altered the estrous cycle toward a normal cycle. In addition, the weight gain of the offspring-PCOS + APN group tended to be normal during the growth process. Because of the significant effect of adiponectin treatment in early pregnancy, we believe that the adiponectin treatment process occurs in the uterus during the embryonic stage. However, the APN level in female adult mice in the offspring-PCOS group was slightly higher than that in the offspring-control group, which contradicts the detected serum APN levels in early pregnancy as well as prepuberty and puberty offspring, as reported by Sir-Petermann et al. [42]. Some studies have shown that pregnant women and fetuses have relatively independent adiponectin regulatory systems. Adiponectin cannot directly pass through the placental barrier, and offspring are not affected by maternal adiponectin levels [43]. This conclusion confirms that there is no correlation between the APN level of offspring mice in adulthood and that of maternal mice in early pregnancy. Some reports also confirmed that a compensatory mechanism of metabolic disorders occurs in PCOS patients, as oxidative stress markers, such as superoxide dismutase and catalase, were detected in the normal range in women with PCOS [44]. In the current study, some offspring may exhibit a series of compensatory effects, the APN level in female adult mice in the offspring-PCOS group was slightly higher than that in the offspring-control group, which is also reflected in the high-density lipoprotein cholesterol concentration in the serum of female offspring rats. We speculate that the significant effect of adiponectin treatment during pregnancy to prevent PCOS-like traits in offspring is most likely to be achieved by normalizing the maternal metabolism and reducing adverse exposure of offspring, rather than via a direct effect of adiponectin on the offspring.

In this experiment, PCOS maternal hypoadiponectinemia was effectively corrected, while PCOS maternal hyperandrogenemia was only improved to a great extent, showing that the androgen level of PCOS pregnant mice during pregnancy was still slightly higher than that of normal mothers after APN intervention. This means that offspring of female mice were still exposed to a low-dose androgen environment during the embryonic period. Studies have shown that prenatal androgen exposure has a dose-dependent effect on the metabolism of offspring in adulthood [45]. On the one hand, low-dose androgen exposure can enhance the expression of steroidogenic acute regulatory protein (StAR) and peroxisome proliferator-activated receptors γ (PPARγ), which regulate the utilization of cholesterol, showing an antioxidant response in PCOS offspring; on the other hand, high-dose androgen exposure can induce a pro-inflammatory state in ovarian tissue mediated by a high prostaglandin level and the expression of cyclooxygenase-2 protein (COX2). In this study, we speculated that APN intervention in early pregnancy did not directly cut off the fetal androgen exposure of PCOS mice but transformed unfavorable high-dose exposure into favorable low-dose exposure, but this conjecture still needs to be verified by further experiments.

The aim of this study was to examine whether exogenous adiponectin supplementation in early pregnancy is necessary for normalizing the endocrine phenotype of adult female PCOS offspring mice. We detected the relative expressions of phosphorylated AMPK, PI3K, and Akt in the mature ovaries of PCOS offspring pretreated with or without APN during the embryonic stage and found that the AMPK/PI3K-AKT pathway was activated in the offspring-APN + PCOS group. By correlating the levels of adiponectin in each offspring, we can conclude that the AMPK-mediated PI3K/AKT pathway is activated in adiponectin-treated offspring mice; this pathway consumes adiponectin in the circulation and decreases the adiponectin level to its normal value. However, the low expression of the AMPK/PI3K-AKT pathway in PCOS offspring has undergone irreversible programming during formation in the embryonic stage [46, 47], and the primary dysfunction of ovarian tissue in offspring leads to the loss of its function of compensating elevated APN levels. This process leads to the accumulation of some unmetabolized APN in the peripheral circulation, resulting in a transient increase in serum APN levels. This conclusion answers the opposite result of serum adiponectin levels in the two generations of maternal and offspring, and further reflects that taking measures to eliminate adverse factors during embryogenesis is of great significance to improve the offspring’s overall metabolic function. On the other hand, we can see that the offspring body’s compensation mechanism for high androgen exposure during the embryonic stage is also achieved by increasing peripheral adiponectin levels, which reflects the sensitivity of APN in metabolic and reproductive regulation in PCOS patients. As a result of these data, we infer that adiponectin supplementation during the embryonic period improved the metabolic syndrome of PCOS offspring in adulthood via activation of the AMPK/PI3K-Akt pathway.

## Conclusions

Our current study provided new data on the relationship between exogenous APN supplementation and the metabolic consequences for offspring with prolonged androgen and hyperlipidemia exposure in the embryonic period. As adiponectin works, treatment is most likely to be achieved by normalizing the metabolism of the mother and reducing the adverse exposure of the offspring. We speculated that compared with the direct treatment of metabolic disorders of PCOS offspring in adulthood, more offspring may benefit from early pregnancy treatment. This is supported by the fact that for PCOS patients with abnormal body mass (obesity) and high androgen levels, APN supplementation during the first trimester of pregnancy is an effective preventive strategy to correct the metabolic disorders of female offspring in adulthood. However, adverse drug reactions and the use-value of this program in clinical practice still need to be further explored.

## Data Availability

All the data is contained in the manuscript.

## Abbreviations

AF: Antral follicle
Akt/PKB: Protein kinase B
AMH: Anti mullerian hormone
AMPK: Adenosine monophosphate activated protein kinase
APN: Adiponectin
CAMKK2: Calcium/calmodulin dependent protein kinase kinase2
CF: Cystic follicle
CL: Corpora lutea
COX2: Cyclooxygenase-2 protein
DHEA: Dehydroepiandrosterone
FBG: Fasting blood glucose
GC: Granulosa cell
GSK3β: Glycogen synthase kinase 3β
HCG: Human chorionic gonadotropin
HDL-C: High density lipoprotein cholesterol
HOMA-IR: Homeostasis model assessment-insulin resistance
INS: Insulin
I.p: Intraperitoneally
IPGTT: Intraperitoneally glucose tolerance test
IPITT: Intraperitoneally insulin tolerance test
IRS-1: Insulin receptor substrate-1
LDL-C: Low density lipoprotein cholesterol
PCOS: Polycystic ovary syndrome
PF: Preovulatory follicle
PI3K: Phosphatidylinositol 3-kinase
PMSG: pregnant mare serum gonadotropin
PPARγ: Peroxisome proliferator-activated receptors γ
qRT‐ PCR: Quantitative real‐ time PCR
SHBG: Sex hormone-binding globulin
S6K1: Ribosomal protein S6 kinase 1
StAR: Steroidogenic acute regulatory protein
T: Testosterone
TC: Total cholesterol
TG: Triglycerides
